# Lacrimal gland choristomas

**DOI:** 10.5935/0004-2749.20220029

**Published:** 2025-08-21

**Authors:** Antonio Augusto V. Cruz, Roberto Murillo Limongi, Eduardo Damous Feijó, Tim Jürg Enz

**Affiliations:** 1 Department of Ophthalmology, Faculdade de Medicina de Ribeirão Preto, Universidade de São Paulo, Ribeirão Preto, SP, Brazil; 2 Department of Ophthalmology, Universidade Federal de Goiás, Goiânia, GO, Brazil; 3 Department of Ophthalmology, Cantonal Hospital Aarau, Aarau, Aargau, Switzerland

**Keywords:** Lacrimal gland, Choristoma, Prognosis, Glândula lacrimal, Coristoma, Prognostico

## Abstract

The purpose of this article was to report a case of intraconal lacrimal gland tissue and to review the literature on lacrimal gland choristoma. The magnetic resonance imaging findings of a biopsy-proven orbital case are also presented. A PubMed database search was performed using the key terms heterotopic, ectopic, aberrant, choristoma, and lacrimal gland to identify all the previously documented studies on lacrimal gland choristoma, in English, Spanish, and French. We classified the lacrimal gland choristoma cases classified according to the location of the lesions, clinical appearance, management, and outcome. The search targeting the period between 1887 and 2019 returned 79 articles, which were reviewed. We found a total of 113 cases of choristomas with normal lacrimal gland tissue. Only two of them were not associated with the eye or its adnexa while the remaining 111 lesions were found either on the ocular surface (n=46) or in the orbit (n=34). Intraocular choristomas were found in 18 patients, and the rest of the lesions were noted either on the eyelids (n=10) or in the lacrimal drainage system (n=3). Orbital and intraocular choristomas are the most harmful lesions as orbital choristomas are frequently associated with permanent diplopia while intraocular lacrimal gland choristomas have a poor visual prognosis and are a common cause of enucleation of the eye. In one of the reported cases, a corneal lacrimal gland choristoma had been experimentally induced by activating the FGF10 signaling pathway. Lacrimal gland choristomas are not uncommon. This peculiar type of lesion has been experimentally induced and may appear in a variety of locations associated with the globe and its adnexa.

## Abstract (pt)

Descrever um caso de tecido de glândula lacrimal orbitário intracônico e revisar a literatura sobre choristomas lacrimais. Os termos heterotópico, ectópico, aberrante, choristoma e glândula lacrimal foram usados para procurar manuscritos em inglês, espanhol e francês. Todos os artigos encontrados foram revisados. Os choristomas lacrimais relatados foram classificados segundo a localização das lesões, aparência clínica, manejo e desfecho. Imagens de ressonância magnética de um novo caso orbitário comprovado por biópsia também são apresentadas. Foram revisados setenta e nove artigos, de 1887 a 2019, descrevendo 113 casos de choristomas constituídos por tecido de glândula lacrimal normal. Apenas 2 casos (2%) não estavam associados com o olho ou seus anexos. A maioria das 111 lesões restantes foram encontradas na superfície dos olhos (n=46) e nas órbitas (n=34). Os choristomas intraoculares foram encontrados em 18 pacientes. As demais lesões foram diagnosticadas nas pálpebras (n=10) e no sistema lacrimal (n=3). Os choristomas orbitais e intraoculares foram as lesões mais deletérias. Na órbita eles são frequentemente associados com diplopia permanente. Os choristomas lacrimais intraoculares imitam neoplasias e são uma causa comum de enucleação. Os choristomas lacrimais não são incomuns. Este tipo peculiar de lesão já foi induzido experimentalmente uma única vez e pode aparecer em uma variedade de locais associados ao globo e sua anexos. Os choristomas intraoculares têm um prognóstico visual ruim.

## INTRODUCTION

Lacrimal gland choristomas are benign lesions formed by normal lacrimal gland tissue (LGT) present outside the lacrimal fossa. LGTs may occur in a variety of unusual places and are either heterotopic^(1)^, aberrant^(2)^, or ectopic^(3)^. This type of choristomas is uncommon and extremely difficult to identify in a clinical setting as they may mimic neoplastic^(4)^ or parasitic lesions^(5)^. The knowledge related to these lesions is limited due to the inconsistency in the terminologies used to describe them. Also, the information pertaining to its epidemiology, topographic location, and neoplastic transformation is unorganized and scattered across the literature, adding to the challenge.

The purpose of this article was to describe a new case of intraconal LGT and perform a literature review of similar previous studies that were based on the location and clinical characteristics of the lacrimal gland choristomas.

## METHODS

We searched the PubMed database using the key terms heterotopic, ectopic, aberrant, choristoma, and lacrimal gland (LG), for articles published between 1887 and 2019. All articles in English, Spanish, and French were reviewed and categorized based on the location of the lesions, their clinical appearance, management, and outcome. We also screened the references cited in the articles for additional reports, which were included in the literature analysis.

## RESULTS

We found 79 articles from 1887 to 2019, describing 113 cases of LG choristomas^(1-78)^. LGT was reported outside the limits of the eye and its adnexa in only two cases. As shown in tables 1 to 4, the remaining 111 cases were associated with either the eyeball (n=64%, 57.6%)^(1-4,6-12,14,20,23,24,26,27,29,30,33,34,38,40,43,45-47,49,50, 54-56,58,61,66,69,71,74,76,79)^, orbit (n=34, 30.6%)^(5,12,13,15-18, 21,22,25,32,35-37,42,48,51,57,59,60,68,70,73,75,77,78)^, or less commonly, the eyelids (n=10, 9.0%)^(31,39,41,52,53,65,72,73)^. Out of the 64 cases related to the globe,18 (28.1%) were intraocular uveal choristomas^(2-4,10,12,14,20,23,30,33,34,38,40,45-47,49,69)^ and 46 (71.9%) were epibulbar lesions^(1,7-9,11,24,26,27,29,43,50,54-56,58,61,71,74,76,79)^. Interestingly, 38 (82.6%) of the 46 epibulbar lesions were found in the lateral half of the globe^(1,6,7,11,26,27,29,43,50,54,56,66,71,74,76,79)^, involving the superotemporal quadrant^(1,11,26,27,29,43,66,71,74,76)^ in majority of the cases (n=15, 32.6%). Among the 34 orbital choristomas, 16 (50%) were diagnosed on the lateral aspect of the intraor extraconal orbital compartment^(12,13,15,16,18,22,25,32,42,48,57,60,67,73,75)^. Kural, in his article, described the case of a 9-year-old boy with a lacrimal fistula in the middle of the temporal fossa, which was surgically explored and an extraorbital LG was removed^(28)^. Pe’er reported the finding of normal LGT in the nasal mucosa, in his study^(19)^. The occurrence of ectopic LGT within the lacrimal drainage system is extremely rare with only three cases reported in the lacrimal sac so far^(62,63,65)^.

**Table 1:** Epibulbar lacrimal choristomas

Author/year	Cases	Sex	Age	Location	Clinical appearance	Treatment	Histopathology
Dame/1946^(6)^	1	M	28 years	Inferotemporal bulbar	Mass	Excision	Normal LGT
				conjunctiva/Cornea			
François et al./l 951^(7)^	1	F	36 years	Inferonasal limbal conjunctiva	Cystic	Excision	Adenoma admixed with normal LGT
Braun-Vallon/1955^(74)^	1	F	8 years	Superotemporal epibulbar	Mass	Excision	Normal LG
Boase/1954^(8)^	1	F	30 years	Medial bulbar conjunctiva	Mass	Excision	LG adenoma
Allende/1954^(29)^	1	M	7 years	Superotemporal bulbar	Mass	Biopsy	Normal LGT
				conjunctiva/Cornea			
Hughes et al/1956^(56)^	1	F	23 years	Inferotemporal Limbus/Cornea	Mass	Excision	Normal LGT
Mettier/1958^(9)^	1	F	21 years	Inferonasal Cornea/Sclera	Nodular	Excision	Normal LGT
Gõrdüren/1962^(1l)^	1	M	6 years	Superotemporal bulbar	Nodular	Excision	Glandular acini + fibrosis
				conjunctiva			
Kessing/1968^(58)^	1	F	23 years	Inferonasal limbus	Mass	Excision	Lacrimal tissue
Pfaffenbach et al/1971^(66)^	2	F	7 years	Superotemporal and superonasal	Mass	Biopsy	Lacrimal tissue
				bulbar conjunctiva/cornea OU			
		M	13 months	Superotemporal bulbar	Mass	Excision	Lacrimal tissue
				conjunctiva			
First et al/1971^(76)^	2	F	11 years	Superotemporal bulbar	Cystic	Excision	Lacrimal tissue
				conjunctiva			
		M	31 years	Superotemporal bulbar	Cystic	Excision	Lacrimal tissue
				conjunctiva			
Bullock et al/1986^(24)^	1	M	66 years	Left medial canthus	Cystic	Excision	Multiloculated cyst with islands of LGT
Hered et al/1987^(26)^	1	F	6 months	Superotemporal episcleral	Mass	Excision	Small nests of ectopic LGT underlying a dermolipoma and osteous choristoma
Pokorny et al/1987^(27)^	3	M	16 years	Superotemporal limbus and	Mass	Incisional	Multiple lobules of well-formed
				cornea		biopsy	lacrimal tissue, associated with loose fibroadipose tissue, smooth muscle
							and cartilage
		M	9 months	Inferotemporal limbus and cornea	Nodular	Excisional	Multiple lobules of well-formed
						biopsy	lacrimal tissue, associated with loose
							fibroadipose tissue
		F	13 years	Superotemporal limbus and	Mass	Partial	Well differentiated LG lobules
				cornea		resection	associated with hyaline cartilage and
							smooth muscle
Kim et al/1989^(1)^	8	F	5 years	Lateral bulbar conjunctiva	Mass	NS	LGT associated with fat, fibrosis and
							smooth and skeletal muscle
		F	15 years	Superonasal bulbar conjunctiva	Mass	NS	LGT associated with fat, fibrosis and
							smooth and skeletal muscle
		F	10 years	Superotemporal bulbar	Mass	NS	LGT associated with pilosebaceous
				conjunctiva			elements fibrosis and smooth and
							skeletal muscle and nerves
		M	12 years	Lateral bulbar conjunctiva	Mass	NS	LGT associated with sweat glands, pilosebaceous elements fibrosis and
							smooth and skeletal muscle
		M	19 years	Nasal bulbar conjunctiva	Mass	NS	LGT
		M	14 years	Lateral bulbar conjunctiva	Mass	NS	LGT with pilosebaceous components, fibrosis, smooth muscle and nerves
		M	2 years	Inferotemporal limbus	Mass	NS	Lacrimal tissue with fibrosis
		M	5 years	Lateral bulbar conjunctiva	Mass	NS	LGT associated with fat, fibrosis and
							smooth and skeletal muscle
Rao et al/1989^(54)^	1	M	40 years	Lateral bulbar conjunctiva	Cystic	Excision	Lacrimal duct cyst
Duncan et al/1998^(43)^	3	F	2 years	Superotemporal limbal	Mass	Excision	Complex choristomas containing
							lacrimal tissue
		F	9 months	Superotemporal and	Mass	Excision	Complex choristomas containing
				inferotemporal limbal			lacrimal tissue
		M	27 months	Bilateral limbal: Superotemporal,	Mass	Excision	Complex choristomas containing
				inferotemporal and nasal			lacrimal tissue
Patyal et al/2010^(50)^	1	F	13 years	Lateral fornix	Mass	Excision	Pleomorphic adenoma
Raina et al/2010^(71)^	1	M	1 months	Bilateral bulbar conjunctiva. Supero	Mass	Biospy	Normal glandular tissue
				and inferotemporal, superior			
Ferri et al/2013^(55)^	1	F	25 years	Inferomedial bulbar conjunctiva	Mass	Excisional	Dermolipoma + LGT
						biopsy	
Raven et al/2016^(61)^	1	M	55 years	Caruncle	Mass	Excision	LG acini
Aldossary et al/2018^(79)^	12	12 out of		Temporal in most cases	Mass	Excision	LG acini
		15 complex choristomas					
Total of cases	46, 34 patients with age and sex specified=16 Males/18 Females						

**Table 4:** Eyelid lacrimal choristomas

Author/year	Cases	Sex	Age (years)	Location	Clinical findings	Imaging	Treatment	Histopathology	Outcome
Evans/1964^(72)^	1	F	50	Lower eyelid	Firm mass on nasal aspect of the lower lid	None	Excision	Pleomorphic adenoma	NS
Jain/1964^(73)^	1	NS	48	In the middle of the upper eyelid	Nodular mass/ Mechanical ptosis	None	Excision	Pleomorphic adenoma	NS
Gordon et al/1991^(31)^	1	F	2	Upper eyelid	Ectopic cilia and lacrimal fistula	None	Excision	Lobules of LG associated with ectopic cilia	Cured
Sim et al/1999^(39)^	1	F	15	Upper eyelid	Watering fistula	Gallium-67 uptake	Excision	Intratarsal lacrimal tissue	Cured
Lee et al/2002^(41)^	1	F	58	Upper eyelid OU	Nodular tarsal areas	None	Tarsectomy	Lacrimal tissue	Cured
Alsuhaibani/2012^(52)^	1	F	75	Upper eyelid	Upper eyelid swelling	CT	Excision	Pleomorphic adenoma	Cured
Obi et al/2013^(53)^	3	M	12	Medial lower eyelid	Mass	None	Excision	Pleomorphic adenoma	Cured
		M	52	Medial lower eyelid	Nodular lesion	None	Excision	Pleomorphic adenoma	Cured
		M	64	Central lower eyelid	Mass	None	Excision	Pleomorphic adenoma	Cured
Wajda et al/2019^(65)^	1	F	35	Upper eyelid	Mass	CT	Excision	Pleomorphic adenoma	NS
Total	10, 4 Males/5 Females/1 Not stated								

The overall female-to-male ratio was 1:1. The age of the patients at the time of surgical management of the LG choristomas correlated with the location of the lesion. The intraocular choristomas were managed at a median age of 10.5 months, whereas the epibulbar, orbital, and eyelid lesions were addressed much later in life, at a median age of 13, 18, and 43.5 years, respectively. Orbital choristomas were especially difficult to differentiate from inflammatory and neoplastic conditions. In all the reported cases, the LG choristomas were confirmed only after biopsy and histopathological examination.

### Case Presentation

**Figure 1:**
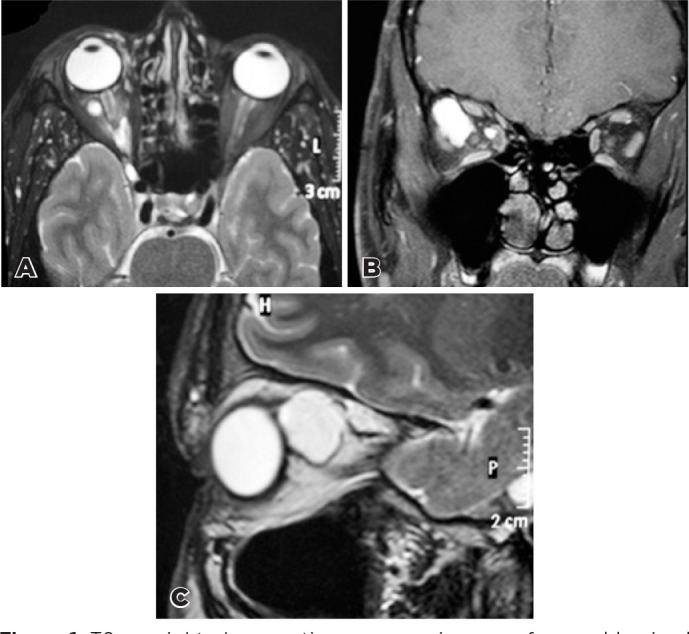
T2 - weighted magnetic resonance images of normal lacrimal tissue within the intraconal space of the right orbit. Notice the isolated tissue lobe in the axial (A) and coronal (B) slices. A large apical component of the lesion is seen in the sagittal slice (C).

**Figure 2:**
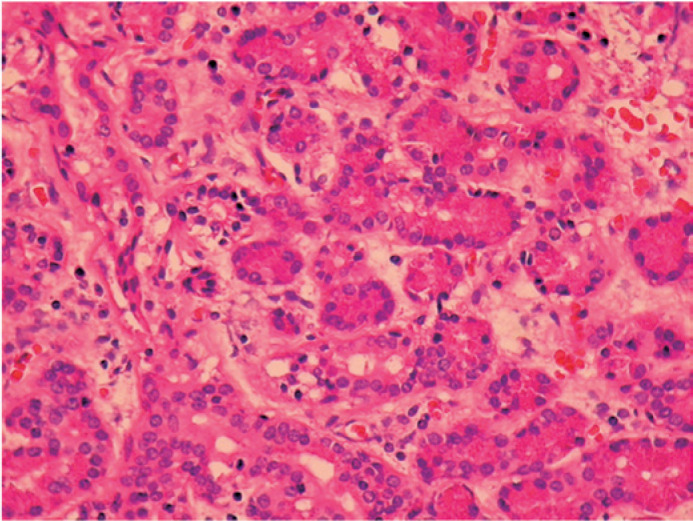
Histopathology showing normal lacrimal gland tissue (Hematoxylin-eosin x 200).

A 30-year-old male presented to the University Hospital of Ribeirão Preto for a follow-up examination after having undergone lateral orbitotomy elsewhere 2 years ago. According to him, the procedure had been performed to treat right eye proptosis, secondary to a large orbital mass. His past medical history was significant for multiple steroid injections into the right orbit for the management of a presumed orbital hemangioma during childhood. Examination of the right eye revealed no proptosis and unrestricted ocular motility. The funduscopic and visual field examinations were normal. Magnetic resonance imaging (MRI) showed multiple, large intraconal cysts in the right orbit. T1- and T2-weighted MRI revealed well-circumscribed masses around the right optic nerve with high signal intensity compared to the vitreous on T1W and T2W images (Figure 1). Histopathological evaluation of the sections of specimen obtained from the previous orbital surgery revealed ducts and acini of benign LGT (Figure 2) and absence of atypical cells, mitotic activity, and necrosis. No further intervention was planned as the patient was asymptomatic.

## DISCUSSION

The occurrence of ectopic LGTs and intraocular lacrimal gland choristomas, as well as their progression into tumors, has been known since the 19th century. In 1891, Schirmer reported a case of conjunctival adenoma located between the insertion point of the medial rectus muscle and plica semilunaris^(7)^. Puech is considered to be the first to have reported a choroidal adenoma in an adult female in 1887^(10,14)^. A large number of LG choristoma cases have been published since these early reports. Although the present review is highly representative of the studies reported in the literature, it is not exhaustive. Some ancient papers could not be retrieved, and manuscripts written in European languages were not included. The analysis of the literature review described in this article is similar to that of a recent publication on the same subject^(81)^ where it has been presented in a more condensed form.

A question is whether ectopic LGT lesions are choristomas. The term choristoma is derived from the Greek word *choristos* (separated)-a tumor-like growth of microscopically normal tissue presents in an abnormal location^(82)^. The LG is located in the orbital lacrimal fossa. Accessory LGs are present in the superior and inferior conjunctival fornix (glands of Krause), along the nonmarginal border of the tarsal plates (glands of Wolfring), and in the plica semilunaris and the caruncle^(83)^. Thus, LGT lesions should be considered choristomas only if found outside these locations. Table 2 presents the cases of intraocular lacrimal choristomas found at different locations, as reported by several authors over the years. Ectopic LGT lesions might be a result of changes in the accessory lacrimal glands as in the case reported by Boase and Bullock who found an “ectopic” lacrimal cyst involving the caruncle or the medial aspect of the conjunctiva near the plica^(8,24)^.

**Table 2:** Intraocular lacrimal choristomas

Anthor/year	Cases	Sex	Age	Location	Clinical appearance	Treatment	Histopathology
Christensen et al/1952^(2)^	1	M	2 weeks	Iris, ciliary body, sclera	Cystic	Enucleation	Cystic glandular implants in the sclera with ducts communicating with subconjunctival space and cyst of the anterior chamber
Bruce/1952^(69)^	1	M	2 months	Iris	Mass	Excision	Normal lacrimal tissue
Hunter/1960^(10)^	1	M	9 months	Iris, ciliary bod, cornea	Cystic	Enucleation	Normal LGT
Dallachy/1961^(3)^	1	F	9 months	Iris, ciliary body	Nodular	Excision followed by enucleation	Normal LGT
Green et al/1967^(12)^	1	NS	11 months	iris, ciliary body	Nodular	Enucleation	Normal glandular tissue
Morgan et al/1972^(14)^	1	M	9 months	Ciliary body	Cystic	Enucleation	LGT in the ciliary body. Cyst of LGT within the anterior chamber
Conway et al/1985^(20)^	1	F	22 months	Ciliary body, choroid, superficial sclera	Mass/Cystic	Enucleation	LG choristoma
Ghadially et al/1986^(23)^	1	F	11 months	Ciliary body, choroid, limbal surface and anterior sclera	Cystic	Enucleation	Normal lacrimal tissue
O’Donnell et al/1990^(30)^	1	M	19 months	Iris	Nodular	Biopsy	Normal LG
Shields et al/1995^(33)^	1	F	19 months	Iris, ciliary body	Cystic	Iridocycletomy	Glandular mass with an irregular cystic structure
Rowley et al/1997^(34)^	1	M	5 years	Iris, epibulbar	Cystic/Mass	Incisional biopsy	Ectopic LGT
Kluppel et al/1999^(38)^ Shields et al/2000^(40)^	1 1	F M	2 months At birth	Iris Iris, ciliary body	Cystic Mass	Excision Iridocyclectomy	Lacrimal serous gland tissue Benign LGT
Cho et al/2006^(45)^	1	F	10 months	Ciliary body	Cystic/mass	Partial sclerocyclectomy	Normal LG with cystic spaces
Jung et al/2006^(46)^	1	F	2 months	Choroid	Cystic	Incisional biopsy	Ectopic lacrimal tissue
Kobrin et al/2007^(47)^	1	M	6 years	Iris	Mass	FNAB	Features characteristic of a benign glandular epithelium consistent with LG acini
Ranganathan et al/2010^(49)^	1	F	6 weeks	Iris/ciliary body	Mass/Multi cystic	Enucleation	LGT with dilated ducts
Tauziède-Espariat et al/2016^(4)^	1	F	12 months	Ciliary body	Mass	Iridocyclectomy, enucleation	LGT
Total of cases	18, 8 Males/9 Females/1 Not stated						

The second important characteristic of an ectopic choristoma is that it is distinctly separated from the anatomical structure it is derived from. A definite cleavage between a superolateral mass of LGT and the LG should be evident for consideration of an LGT as ectopic^(84)^. Ectopic LGTs have been noted to be most commonly located in the epibulbar conjunctiva. However, in some of the epibulbar LGT cases reported previously, it is not clear if the superolateral masses that were considered as ectopic were actually detached from the LG^(11,26,27,29,43,54,61,66,71,74,76)^.

A critical analysis of the published cases also raises doubts about whether all the reported lesions within the orbit represent true choristomas. Green et al. described a case wherein the excised tissue was attached to the orbital lobe of the LG^(12)^. Kao reported a case of an inferior dacryops (rare, benign lacrimal gland/duct cyst) that was similar that of the accessory LGs usually found in the inferior fornix^(78)^. The orbital images of superotemporal lesions found in some other cases do not show a clear disunion between the LGTs labeled as ectopic and the LG^(32,60)^.

Only two other studies^(12,85)^ on LG choristomas have described the distribution of lesions similar to that presented in our review. Alyahya et al analyzed sections of 61 LG choristoma specimen spanning 50 years, collected from the files of the Eye Pathology Institute of Copenhagen. They compared the details on location and clinical diagnosis of the lesion specimen with their histological findings. Out of the 61 lesions, 43 (70.5%) were found on the superotemporal portion of the bulbar conjunctiva^(85)^. Although these studies did not provide any clinical information, they clearly specified the location of the lesions.

Knowledge of the LG’s embryonic development is essential for speculating about the origin of lacrimal gland choristomas. LG formation consists of three stages^(86)^. During stage 1, LG appears as a thickening of the superior fornix epithelium and surrounding mesenchymal condensation at O’Rahilly stages 19-20 (crown-rump length 17-20 mm, 48-51 days). Stage 2 marks an epithelial bud formation at O’Rahilly stages 21-23 (crown-rump length 23-28 mm, 52-57 days). Stage 3 of LG development occurs during the fetal period (weeks 13-16) and is characterized by gland maturity.

The embryonic development of LG is based on the general process of branching morphogenesis as it has a tubulo-acinar branching structure (lobules formed by acini and intralobular, interlobular, and excretory ducts) similar to a bunch of grapes^(87,88)^. LG development depends on the interaction between the surface ectoderm and surrounding mesenchyme, which is facilitated by the exchange of chemical signals between groups of cells. This occurs through specific signaling pathways such as fibroblast growth factor 10 (FGF10), homeobox transcription factor Barx2, bone morphogenetic protein 7 (BPM7), and the canonical Wnt signaling family^(89-91)^.

Mesenchymal FGF10 expression is crucial for LG induction^(92-94)^ as it promotes epithelial gland proliferation by directly activating FGF receptor-2 IIIb in the conjunctival epithelium. Barx2, BPM7, and the canonical Wnt signaling family are responsible for the branching morphogenesis. Barx2, which is expressed in the epithelium, is necessary for branching elongation^(95)^. BMP7 is expressed mainly in the mesenchyme, where it promotes the proliferation and aggregation of the cells, and a lack of this molecule impairs bud formation^(96)^. The canonical Wnt cascade, on the contrary, is induced in the gland epithelium, inhibiting branching^(97)^.

The evolving knowledge of the molecular mechanisms involved in LG morphogenesis rules out the old theory that lacrimal gland choristomas are a result of an aberrant migration of cells^(2)^. In fact, it has already been experimentally demonstrated that activation of the FGF10 signaling pathway can induce LGT formation within the cornea^(94)^. Thus, it can be safely concluded that lacrimal gland choristomas result from localized abnormal expression of the signaling molecules associated with LG morphogenesis. A similar conclusion was drawn by Milman et al. who analyzed the immunohistochemical characteristics of lacrimal hamartomas^(98)^. They also noted that these lesions are derived from indigenous conjunctival precursor cells activated by mesenchymal influences.

The clinical management of lacrimal gland choristomas and their impact on vision depend on the location of the LGT. Epibulbar lesions can be easily excised without serious impairment of visual function. Choristomas in the eyelids also present as solitary masses which can be easily managed. However, orbital choristomas are very difficult to diagnose and remove and can severely impair vision. As noted in table 2, 8 out of 34 patients had persistent diplopia^(12,13,15,48,59,75,77)^, two patients suffered vision loss^(12)^, and one patient had to be exenterated due to malignant transformation^(36)^.

Intraocular development of LGT poses the highest risk for vision loss. Based on the information in the literature, 9 out of 18 affected eyes were enucleated^(2-4,10,12,14,20,23,49)^, mainly due to the difficult differential diagnosis of intraocular malignancies such as medulloepithelioma^(4)^. The tear fluid is composed of electrolytes, metabolites, lipids, mucins, and a large number of proteins. Recent proteomic studies have identified more than 1,516 proteins in tears^(99)^. Therefore, intraocular tear fluid production by ectopic LGT could contribute to the inflammation^(34)^ or increase in ocular pressure, which has been verified in several studies^(14,30,33,38,47,49)^.

## Figures and Tables

**Table 3:** Orbital lacrimal choristomas

Author/year	Cases	Sex	Age (yrs)	Location	Clinical findings	Imaging	Treatment	Histopathology	Outcome
Jain/1964^(73)^	1	M	14	Superotemporal extraconal	Proptosis	X-Rays	Excision	Pleomorphic adenoma	NS
Boudet et al/1964^(75)^	1	F	61	Inferotemporal intraconal	Proptosis/diplopia	Orbital angiography	Excision	Pleomorphic adenoma	Cured/diplopia on downgaze
Bech et al/1965^(57)^	1	M	50	Inferonasal orbital rim	Palpable mass	None	Excision	Pleomorphic adenoma	Cured
Green et al/1967^(12)^	8	F	45	Extraconal, medial wall	Proptosis, vertical dystopia	None	Excision	Atrophic lacrimal tissue with	Favorable
								moderate chronic	
								inflammatory	
								infiltrate	
		M	44	Extraconal lateral wall.	Proptosis, diplopia	X-ray	Excision	Atrophic lacrimal	Persistent
				Attached to the orbital				tissue with fibrous	diplopia
				lobe of the lacrimal gland				stroma	
		F	16	Extraconal superonasal.	Proptosis, diplopia,	None	2 Biopsies	Autopsy after	Accidental car
				Normal lacrimal tissue/	palpable mass			accidental death:	death
				Adenocarcinoma				ectopic LGT with	
								cysts, areas of	
								adenocarcinoma	
		F	42	Extraconal	Retrobulbar pain,	None	Excision	Lobules of	NS
				inferotemporal and	limitation of motility,			glandular tissue	
				orbital floor l	Proptosis			with lymphocytic	
								infiltration	
		F	11	Beneath the superior	Ptosis, proptosis,	X-ray	3 Biopsies	Intraconal lesion	Vision loss,
				orbital rim between the	limitation of motility.			with lobules of	proptosis,
				LG and the trochlea.	Palpable mass			LG with acute	eye motility
				Second biopsy:	beneath the superior			and chronic	limitation,
				intraconal	orbital rim			inflammation	corneal clouding
		M	15	Intraconal	Proptosis, vision loss	US	Excision	Glandular tissue	Optic nerve
				superotemporal				lymphocytic	atrophy
								infiltration,	
								fibroadipose	
								connective tissue	
		M	46	Apical extraconal tissue	Proptosis, slightly	Arteriography	2 Biopsies	LGT with cystic	Proptosis
				from a large tumor	reduced acuity			areas	reduction
				involving the LG					
		M	11	Extraconal posterior	Proptosis, vision loss,	US	Biopsy	Lobules of LGT with	Residual
					choroidal folds			moderate atrophy	exophthalmos
								and chronic	
								inflammatory	
								infiltrate	
Baldrige et al/1970^(13)^	1	M	18	Extraconal lateral from the rim to the apex	Proptosis, esotropia	X-ray	Excision	Normal lacrimal gland tissue	Residual esotropia
Zilkha/1972^(15)^	1	F	46	Extraconal lateral	Proptosis, diplopia, adduction limitation	X-ray, carotid angiography,	Excision	Normal LGT with lymphocytic	Residual proptosis,
						orbital		infiltration	diplopia
						venography			improvement
Mindlin et al/1977^(70)^	1	M	21	Extraconal medial	Proptosis, choroidal folds	X-ray, US, orbital arteriography	Excision	Pleomorphic adenoma	Residual macular changes
Jacobs et al/1977^(77)^	1	F	69	Intraconal inferolateral	Proptosis, visual loss, diplopia	CT	Excision	Normal GLT	Diplopia
Müeller et al/1979^(16)^	1	M	31	Intraconal lateral to the optic nerve	Proptosis, choroidal folds, slight visual	X-ray	Excision	Normal glandular tissue + areas	Cured
					acuity loss			of pleomorphic	
								adenoma	
Rush et al/1981^(17)^	1	M	7	Extraconal superonasal	Fullness below the superior medial	CT	Excision	Cyst containing normal LG	NS
					orbital rim				
Appel et al/1982^(18)^	1	M	63	Extra-and intraconal lateral	Proptosis, optic atrophy	CT	Biopsy	Normal LGT + varix	NS
Margo et al/1985^(22)^	1	M	28	Extraand intraconal superolateral	Proptosis	CT	Incisional biopsy	Lacrimal tissue adjacent to the	Favorable
								lacrimal fossa and	
								deep in the orbit	
V. Domarus/1987^(25)^	1	F	2	Extraconal lateral	Proptosis, medial dystopia	CT	Excision	LG cyst	Cured
Guy et al/1989^(68)^	2	M F	1.3 0.5	Intraconal (superior, lateral and medial) Intraconal	Proptosis Proptosis, exposure	CT CT	Biopsy Biopsy	Lacrimal gland tissue Lacrimal gland	NS NS
					keratitis			tissue	
Bocato et al/1991^(32)^	1	F	68	Extraconal superolateral	Proptosis	CT/ US	FNAB	Lobules of ectopic lacrimal gland	Stable
Sakurai et al/1997^(35)^	1	M	9	Extraconal superior	Proptosis, Restricted eye motility, Increased intraocular pressure	CT/ MRI/SPECT/ PET scan	Incisional biopsy	Normal lacrimal gland tissue	Stable
Shields et al/1997^(36)^	1	M	26	Extraconal superonasal	Subcutaneous swelling superonasal	CT/MRI	Biopsy	Adenoid cystic carcinoma	Exenteration
West et al/1997^(37)^	1	F	20	Inferomedial	Hypertropia	CT	Excision	Cyst with lacrimal gland tissue	Cured
Suneetha et al/1999^(67)^	1	M	12	Extraconal Lateral rectus/ periorbita	Adduction Limitation	CT/US	Excision	Nodular lesion with lacrimal gland tissue	Cured
Kao et al/2000^(78)^	1	M	33	Extraconal anterior infero-medial	None	CT/US	Excision	Dacryops	Cured
Solaroglu et al/2005^(42)^	1	M	4	Extraconal lateral	Proptosis, orbital pain. Restricted eye motility	CT	Excision	LG lobules	Favorable
Yüceer et al/2008^(48)^	1	F	5	Intraconal lateral	Proptosis, Restricted eye mobility	CT/MRI	Incisional biopsy by transcranial approach	Normal LGT	Lateral rectus paresis
Lenzi et al/2012^(51)^	1	M	61	Intraconal adherent to medial rectus muscle	Diplopia	MRI	Excision	Cyst of ductal origin	Cured
Braich et al/2014^(59)^	1	F	1.1	Extraconal inferonasal	Cystic lesion adduction limitation Exotropia	MRI	Excision	Ectopic LGT with cysts	Persistent adduction limitation
Pujari et al/2015^(5)^	1	F	15	Cyst adherent to the Inferior rectus muscle	Progressive proptosis and diplopia	US/CT	Excision	LG cyst	Cured
Misra et al/2016^(60)^	1	M	60	Proptosis	CT	Excision	Pleomorphic adenoma	Cured
Total of case	34, 20 Males/14 Females								
